# The complete mitochondrial genome of the axillary seabream, *Pagellus acarne* (Perciformes: Sparidae)

**DOI:** 10.1080/23802359.2018.1450674

**Published:** 2018-04-01

**Authors:** Celestina Mascolo, Marina Ceruso, Giuseppe Palma, Aniello Anastasio, Paolo Sordino, Tiziana Pepe

**Affiliations:** a Department of Veterinary Medicine and Animal Production, University “Federico II”, Naples, Italy;; b Biology and Evolution of Marine Organisms, Stazione Zoologica Anton Dohrn, Naples, Italy;; c Assoittica Italia, Rome, Italy

**Keywords:** *Pagellus acarne*; mitogenomics; Perciformes; Sparidae

## Abstract

The axillary seabream (*Pagellus acarne,* Risso 1827) belongs to the Sparidae family, order Perciformes. This high-valued commercial fish species is distributed along the northern and eastern Atlantic coasts from Norway to Senegal, and throughout the Mediterranean Sea. Its complete mitochondrial genome is 16,486 bp in length, consisting of 13 protein-coding genes, 22 tRNA genes, 2 rRNA genes, and 2 non-coding regions (D-loop, 808 bp and L-origin, 29 bp). Its overall base composition is A: 26,8%, C: 29,0%, G: 17.6%, and T: 26.6%.

The axillary seabream (*Pagellus acarne,* Risso 1827) is a demersal fish belonging to the Sparidae family. Its geographical distribution extends from the Black Sea and the Mediterranean, along the west coast of Europe and Africa, to Norway and Angola (Santos et al. 1995). *Pagellus acarne* is a very common species especially in the Mediterranean, where it is prevalent in commercial and artisanal catches. It is listed as Least Concern in the IUNC Red List of Threatened Species, with the recommendation of reducing current fishing efforts (Russell et al. 2014). In this study, the complete mitochondrial genome of *P. acarne* was sequenced with Illumina HiSeq 2500 System (Illumina, San Diego, CA) (GenBank MG736083). The specimen was collected from the Mediterranean Sea (Gulf of Salerno, Tyrrhenian Sea; FAO area 37; N 40°34′47.40″', E 14°43′56.6'″) and identified based on morphological features. Total DNA was extracted from dorsal fin tissue and stored at the Department of Veterinary Medicine and Animal Production, University “Federico II” (Naples, Italy). The complete sequence was 16,486 bp in length, including 13 protein-coding genes, 2 ribosomal RNA genes (12S rRNA and 16S rRNA), 22 transfer RNA genes (tRNA) and two non-coding regions (D-loop and L-origin). This gene arrangement is similar to the typical vertebrate mitogenome (Wang et al. 2008). Most of the genes were encoded on the heavy strand, while the NADH dehydrogenase subunit 6 (*ND6*) and eight tRNA genes [Gln, Ala, Asn, Cys, Tyr, Ser (*UCN*), Glu, Pro] are encoded on the light strand. The nucleotide composition is A: 26,8%, C: 29%, G: 17.6%, and T: 26.6%, which is similar to other Sparidae mitogenomes (Xia et al. 2008; Shi et al. 2012; Dray et al. 2016). Protein-coding genes began with an ATG start codon, with the exception of *COI* and *ND4* that start with GTG. Four types of stop codons revealed are TAA (*ND1*, *ATP6*, *COIII*, *ND4L*, ND5), AGG (*COI*), T (*COII*, *ND4*, *Cytb*) and TAG (*ND2*, *ATP8, ND6*). The 12S and 16S rRNA genes were located between the tRNA^Phe^ (GAA) and tRNA^Leu^ (TAA) genes, and were separated by the tRNA^Val^ gene as in other vertebrates (Li et al. 2016). The 22 tRNA genes vary from 66 to 73 bp in length. The 808 bp-long control region is located between tRNA^Pro^ (TGG) and tRNA^Phe^ (GAA). The non-coding region (L-strand origin of replication) is 38 bp long and is located between tRNA^Asn^ (GTT) and tRNA^Cys^ (GCA).

The phylogenetic position of *P. acarne* was validated using MEGA6 software (Tamura et al. 2013) to construct a maximum-likelihood (ML) tree with 1000 bootstrap replicates, containing the complete mitogenomes of 10 Sparidae species (Figure 1). The resultant phylogeny shows that the *P. acarne* is closely related to *P. bogaraveo* with high bootstrap value supported. The study of the mitochondrial genome of *P. acarne* may reveal novel barcoding regions and deepen our knowledge of the evolution of sparid fishes.

**Figure 1.**
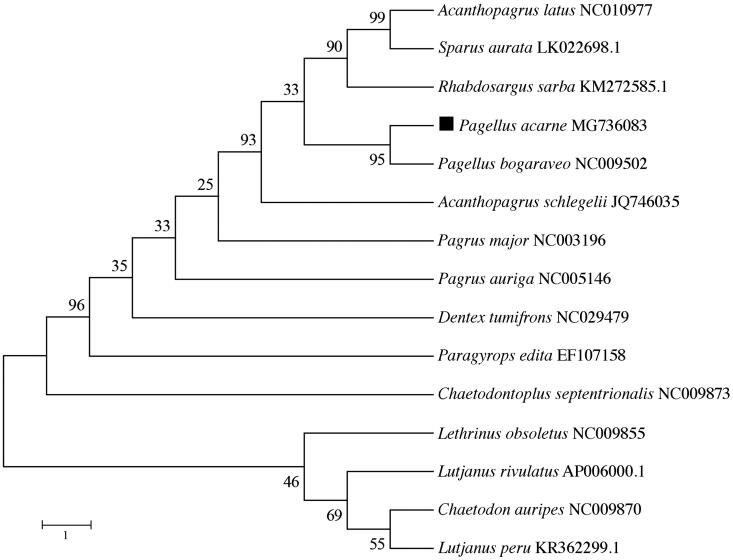
The phylogenetic position of *Pagellus acarne* was validated by ML method with the complete mitogenomes of nine sparids and five arbitrary outgroup species (*Lutjanus peru*, *Lutjanus rivulatus*, *Lethrinus obsoletus*, *Chaetodontoplus septentrionalis*, *Chaetodon auripes*). Numbers above the nodes indicate 1000 bootstrap values. Mitogenome accession numbers are listed behind the species names.
